# Antibiotic prescribing in public primary healthcare centres in Maseru, Lesotho

**DOI:** 10.4102/sajid.v40i1.692

**Published:** 2025-02-12

**Authors:** Mapoloko A. Letša, Johanita R. Burger, Irma Kotzé

**Affiliations:** 1 Medicine Usage in South Africa (MUSA), Faculty of Health Sciences, North-West University, Potchefstroom, South Africa

**Keywords:** AWaRe classification, DDD/100 outpatients/day, antibiotic prescribing patterns, Access-to-Watch index, Amoxicillin Index, Lesotho

## Abstract

**Background:**

Inappropriate prescribing of antibiotics is a global problem. We assessed the prescribing patterns of antibiotics in three public primary healthcare centres (PHCCs) in Maseru, Lesotho.

**Objectives:**

A cross-sectional point prevalence survey was employed using patients’ prescription booklets from October 2022 to December 2022.

**Method:**

Antibiotics were categorised according to the World Health Organization (WHO) AWaRe classification and assessed by Defined Daily Dose (DDD)/100 outpatients/day to measure relative consumption of each antibiotic as a percentage of total consumption, Access-to-Watch index (AW-I) and Amoxicillin Index (A-I).

**Results:**

Of the 624 participants (median age 35 [interquartile range {IQR}: 45–26] years), 71.5% (*n* = 446) were female. Overall mean (standard deviation [s.d.]) antibiotic consumption was 1.48 (0.13) DDD/100 outpatients/day, with PHCC-1 at 1.64, PHCC-2 at 1.33 and PHCC-3 at 1.47 DDD/100 outpatients/day. The median (IQR) AW-I was 4.64 (3.42–9.45) and the A-I was 1.41 (0.87–1.95). The most frequently prescribed Access group antibiotics included amoxicillin (PHCC-2: 45.9%, overall 1.33 DDD/100 outpatients/day; PHCC-3: 24.5%, 1.47 DDD/100 outpatients/day, and PHCC-1: 23.2%, 1.64 DDD/100 outpatients/day) and doxycycline (PHCC-3: 29.9%, 1.47 DDD/100 outpatients/day, 24.1%, PHCC-2: 1.33 DDD/100 outpatients/day). Erythromycin was the most prescribed Watch group antibiotic for all PHCCs.

**Conclusion:**

High consumption of Access-group antibiotics was observed. The Watch group’s antibiotic use, particularly erythromycin, requires the implementation of stewardship programmes. Results may be a baseline for establishing antibiotic stewardship in Lesotho’s PHCCs.

**Contribution:**

Our study addressed the scarcity of data on antibiotic prescribing patterns in PHCCs in Lesotho using the AWaRe classification system recommended for monitoring antibiotic prescribing and promoting rational use.

## Introduction

Antibiotics are the most prescribed medicines in the world, with the level of use increasing over the past decades, particularly in low- to middle-income countries (LMICs).^1,2^ For example, high prescribing of antibiotics was observed in Zambia, Ethiopia and Ecuador for the treatment of upper respiratory infections, particularly at primary healthcare facilities.^3^ Inappropriate use (e.g. antibiotics being prescribed against the standard treatment guidelines) and high prescribing of antibiotics are risk drivers of antibiotic resistance,^4^ which has become a global public health concern.^5,6^ Antibiotic prescribing practices have been shown to be inappropriate in LMICs such as South Africa,^7,8^ Lesotho^9^ and Ghana.^10^

In an attempt to guide healthcare professionals in prescribing antibiotics, the World Health Organization (WHO) developed the Access, Watch Reserve (AWaRe) classification in 2017, which is reviewed every 2 years.^11^ The 2021 WHO AWaRe classification of antibiotics for evaluation and monitoring of use divides antibiotics into four categories: Access, Watch, Reserve and Not Recommended to emphasise the importance of appropriate use. The Access antibiotics, a list of 91 narrow-spectrum antibiotics, can be used as first or second-line therapy for commonly encountered infections and have a low incidence of causing antimicrobial resistance. Examples of Access group antibiotics, among others, include penicillins, aminoglycosides and first-generation cephalosporins. The Watch group, comprising 145 antibiotics, should be prioritised in monitoring by antimicrobial stewardship programmes.^11^ Watch group antibiotics are broad-spectrum antimicrobials with a higher potential to cause antimicrobial resistance. In addition to being listed on the WHO Model Lists of Essential Medicines, they are recommended as essential first or second-choice empiric therapy for a restricted range of infectious syndromes. The Reserve group antibiotics are the last option for treating confirmed multidrug-resistant microorganisms. The Reserve group antibiotics listed on the WHO Model Lists of Essential Medicines are based on their risk-benefit profiles and evidence that they are effective against ‘Critical-Priority’ or ‘High-Priority’ pathogens, such as carbapenem-resistant *Enterobacteriales* and methicillin-resistant *Staphylococcus aureus*. Both critical and high-priority bacteria are resistant to first and second-line antibiotics. Yet, they cause varying degrees of life-threatening illnesses, with critical-priority pathogens causing more severe illnesses than high-priority infections.^12^ To preserve their effectiveness, these antibiotics should be protected, used as a last resort and prioritised as key targets of stewardship programmes. The Reserve category has 31 antibiotics, such as third and fourth-generation cephalosporins and fluoroquinolones. Lastly, the Not-Recommended group is a list of antibiotics not recommended by the WHO because there is no evidence to support their clinical use. The Not-Recommended antibiotics include 107 fixed-dose combinations containing multiple broad-spectrum antibiotics. Examples of the Not-Recommended antibiotics include colistin and polymyxins.^11^ This classification emphasises that appropriate, narrow-spectrum antibiotics from the Access group should be preferred over broad-spectrum antibiotics from the Watch and Reserve groups to limit antibiotic resistance selection and spread.^11^

There is a scarcity of studies assessing antibiotic prescribing patterns in primary healthcare centres (PHCCs) in Lesotho, with no known studies conducted in Maseru’s primary healthcare system. This study, therefore, aimed to assess the prescribing patterns of antibiotics in public PHCCs in Maseru, Lesotho, using the 2021-WHO AWaRe classification.

## Methods

### Study design, setting, and population

A cross-sectional point prevalence survey was conducted at the three PHCCs in Maseru, Lesotho. Maseru district is the largest among the 10 districts and is the capital town of Lesotho. It has an area of 4279 sq. km and a population of 118 355.^13^ Each of the PHCCs has about 150 patient encounters per day.

The study was carried out from October 2022 to December 2022 for a period of 1 month at each PHCC. All adult patients aged 21 years and older (Lesotho’s Ordinance No. 62 of 1829 establishes 21 as the majority age) who received prescriptions for Anatomical Therapeutic Classification (ATC) J01 antibacterials for systemic use and agreed to participate were included.

### Data source and data collection

The data were collected from patients’ prescription booklets, which they take to every appointment to document the consultation and prescribed medicine. The patients’ prescription booklets were examined to collect data on prescribed antibiotics (last written prescription of antibiotics during the study period) during the PHCCs’ operating hours, 08:00 to 16:30, Monday to Friday.

Patient information (age and sex), prescriber, diagnosis, symptoms and antibiotic treatment (active substance, dosage, frequency and duration) were collected using a predesigned data collection tool.

### Data analysis

Descriptive analysis was performed, expressing the results in frequencies and percentages to show the consumption of antibiotics. The mean, standard deviation (s.d.), median and interquartile range (IQR) were calculated for continuous variables. Data were categorised according to the 2021-WHO AWaRe classification system. Antibiotic prescribing was presented using the DDD/100 outpatients/day, the Access-to-Watch index (AW-I) and the Amoxicillin Index (A-I). In computing the DDD/100 outpatients/day, the denominator was the catchment population of the clinics, which was PHCC-1 (*N* = 3274), PHCC-2 (*N* = 5331), and PHCC-3 (*N* = 2876) (M. Koto MoH, personal communication). Further analysis was conducted to assess whether antibiotic prescribing was performed in accordance with the standard treatment guidelines for Lesotho (STGL). The DDD/100 outpatients/day^14^ was calculated according to Equation 1, the AW-I^15^ according to Equation 2 and A-I^16^ according to Equation 3:

$$\text{DDD}/100\text{ outpatients/day}=\frac{\left(\begin{array}{l} \text{Total number of dosage}\\ \text{strength of each antibiotic}\\ {}*\text{dosage unit} \end{array}\right)}{\left(\begin{array}{l} \text{WHO asssigned DDD}\\ {}*\text{duration of study}*\\ \text{number of outpatients} \end{array}\right)}*100$$ [Eqn 1]

$$\text{AWI}=\frac{\text{Number of DDD per patient per day of Access antibiotics}}{\text{Number of DDD per }100\text{ patients per day of Watch antibiotics}}$$ [Eqn 2]

$$\text{AI}=\frac{\text{Number of DDD per }100\text{ patient per day of amoxicillin}}{\text{Total number of DDD per }100\text{ patients per day of J}01\text{ antibiotics}}$$ [Eqn 3]

### Ethical considerations

The study was granted ethical approval on 12 August 2022 by the North-West University Health Research Ethics Committee (NWU-HREC) (approval number NWU-00016-22-S1) and the Ministry of Health Research and Ethics Committee Lesotho (approval ID: ID 70-2022) on 25 June 2022. The Ministry of Health Lesotho (MoH) District Management Team granted permission before data collection by signing a goodwill letter. Before data collection, the research objectives were explained to participants who gave written informed consent. The research was carried out in accordance with the Declaration of Helsinki and national and institutional standards.

## Results

### Demographic characteristics of participants

Of the 624 participants (median age 35 [IQR: 45–26] years) included in the study, most were in the age group > 46 years (27.1%, *N* = 214) in PHCC-1, 27–35 years (26.8%, *N* = 295) in PHCC-2, and 36–45 years (33.0%, *N* = 115) in PHCC-3 (Table 1). The majority of participants in the study (71.5%, *n* = 446) were female. The prescribers at PHCC-1 (*N* = 3) and PHCC-2 (*N* = 2) were general practitioners, whereas PHCC-3 had registered nurse midwives (*N* = 2) as prescribers (Table 1).

**TABLE 1:** Demographic characteristics of participants.

Demographic characteristics	PHCC-1 (*N* = 214)		PHCC-2 (*N* = 295)		PHCC-3 (*N* = 115)	
	*n*	%	*n*	%	*n*	%
**Age groups (years)**						
< 26	53	24.8	74	25.1	29	25.2
27–35	57	26.6	79	26.8	29	25.2
36–45	46	21.5	69	23.4	38	33.0
> 46	58	27.1	73	24.7	19	16.5
**Gender**						
Female	150	70.1	214	72.5	82	71.3
Male	64	29.9	81	27.5	33	28.7

PHCC, primary healthcare centre.

### Antibiotic consumption by DDD/100 outpatients/day

The overall mean (s.d.) antibiotic prescribing was 1.48 (0.13) DDD/100 outpatients/day. The total antibiotic consumption at PHCC-1 was 1.64 DDD/100 outpatients/day, 1.33 DDD/100 outpatients/day at PHCC-2, and 1.47 DDD/100 outpatients/day at PHCC-3 (Table 2). Amoxicillin and clavulanic acid combination was the most prescribed antibiotic in PHCC-1, at 0.73 DDD/100 outpatients/day, compared to 0.17 DDD/100 outpatients/day in PHCC-2. It was not prescribed at PHCC-3. Amoxicillin was prescribed at 0.61 DDD/100 outpatients/day in PHCC-2, compared to 0.38 DDD/100 outpatients/day (PHCC-1) and 0.36 DDD/100 outpatients/day (PHCC-3). Doxycycline accounted for 0.44, 0.32, and 0.23 DDD/100 outpatients/day in PHCC-3, PHHC-2, and PHCC-1, respectively.

**TABLE 2:** Antibiotic consumption in DDD per 100 outpatients per day per primary healthcare centre.

Antibiotic name	PHCC-1		PHCC-2		PHCC-3		Median	IQR†
	DDD per 100 outpatients per day	%	DDD per 100 outpatients per day	%	DDD per 100 outpatients per day	%		
Amoxicillin and clavulanic acid	0.73	44.5	0.17	12.8	-		0.45	0.31–0.59
Amoxycillin	0.38	23.2	0.61	45.9	0.36	24.5	0.38	0.37–0.50
Azithromycin	0.09	5.5	0.003	0.2	0.09	6.1	0.09	0.05–0.09
Ceftriaxone	0.002	0.1	0.01	0.8	0.001	0.07	0.002	0.002–0.006
Ciprofloxacin	0.0005	0.03	0.02	1.5	0.004	0.3	0.004	0.002–0.01
Cloxacillin	0.01	0.6	0.06	4.5	0.02	1.4	0.02	0.02–0.04
Doxycycline	0.23	14.0	0.32	24.1	0.44	29.9	0.32	0.28–0.38
Erythromycin stearate	0.20	12.2	0.04	3.0	0.37	25.2	0.2	0.12–0.29
Metronidazole	-	-	0.09	6.8	0.14	9.5	0.12	0.10–0.13
Nitrofurantoin	-	-	0.005	0.4	-	-	-	-
Phenoxymethylpenicillin	-	-	-	-	0.04	2.7	-	-

Note: Because of small numbers, some DDD per 100 outpatients per day are reported four digits after the decimal point. The total antibiotic consumption was 1.64 DDD/100 outpatients/day (PHCC-1), 1.33 DDD/100 outpatients/day (PHCC-2) and 1.47 DDD/100 outpatients/day (PHCC-3). Hyphens (-) were used to indicate antibiotics not prescribed.

PHCC, Primary Healthcare Centre; DDD, defined daily dose; IQR, interquartile range.

† , Interquartile Range (Q1 Lower quartile, Q3 Upper quartile).

### Antibiotic prescribing according to the Access, Watch Reserve classification

The mean (s.d.) Access group antibiotic consumption was 1.20 (0.14) DDD/100 outpatients/day, while the median (IQR) Watch group antibiotics were 0.29 (0.19–0.36) DDD/100 outpatients/day. The median (IQR) A-I was 1.41 (0.87–1.95) DDD/100 outpatients/day, and the AW–I was 4.64 (1.43–10.57) DDD/100 outpatients /day. The AW-I was highest in PHCC-2 (16.49 DDD/100 outpatients/day), followed by PHCC-1 (4.64 DDD/100 outpatients/day) and PHCC-3 (2.24 DDD/100 outpatients/day). The overall median (IQR) A-I was 1.41 (0.87–1.95), with an A-I of 2.48, 1.41 and 0.33 DDD per 100 outpatients per day for PHCC-1, PHCC-2 and PHCC-3 respectively (Table 3).

**TABLE 3:** Prescribing patterns per primary healthcare centre according to Access, Watch Reserve classification.

AWaRe classification	PHCC-1		PHCC-2		PHCC-3		Mean	s.d.	Median	IQR†
	DDD per 100 outpatients per day	%	DDD per 100 outpatients per day	%	DDD per 100 outpatients per day	%				
Access	1.35	82.3	1.25	94.0	1.01	68.7	1.20	0.14	-	-
Watch	0.29	17.7	0.08	6.0	0.45	30.6	-	-	0.28	0.18–0.37
AW-I	4.64	-	16.49	-	2.24	-	-	-	4.64	3.42–9.45
A-I	2.48	-	1.41	-	0.33	-	-	-	1.41	0.87–1.95

Note: The total antibiotic consumption was 1.64 DDD/100 outpatients/day (PHCC-1), 1.33 DDD/100 outpatients/day (PHCC-2) and 1.47 DDD/100 outpatients/day (PHCC-3).

AWaRe, Access, Watch Reserve; PHCC, primary healthcare centre; DDD, defined daily dose; s.d., standard deviation; IQR, interquartile range; AW-I, Access-to-Watch Index; A-I, Amoxicillin Index.

† , Interquartile Range (Q1 Lower quartile, Q3 Upper quartile).

#### Diseases commonly diagnosed

**FIGURE 1:**
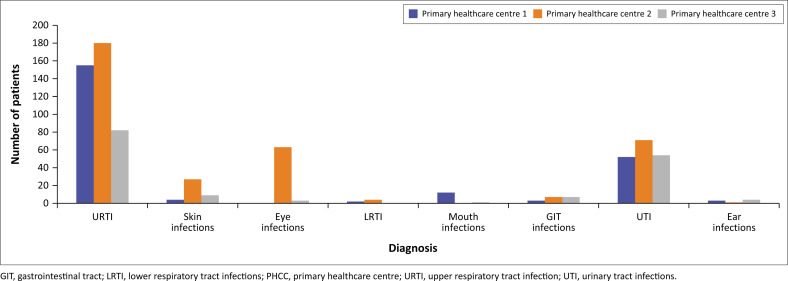
The most prevalent diagnoses made per primary healthcare centre.

A total of 744 diagnoses were recorded, consisting of 231 for PHCC-1, 353 for PHCC-2 and 160 for PHCC-3. The most frequently diagnosed condition in all the PHCCs was upper respiratory tract infection (URTI) (67.1% [*n* = 155], 51.0% [*n* = 180] and 51.3% [*n* = 82] in PHCC-1, PHCC-2 and PHCC-3, respectively). Urinary tract infections (UTI) were the second most recorded diagnosis, at 33.8% (*n* = 54) in PHCC-3, 22.5% (*n* = 52) in PHCC-1 and 20.1% (*n* = 71) in PHCC-2. About 17.8% (*n* = 63) of patients in PHCC-2 were diagnosed with eye infections (Figure 1).

### Commonly prescribed antibiotics

**FIGURE 2:**
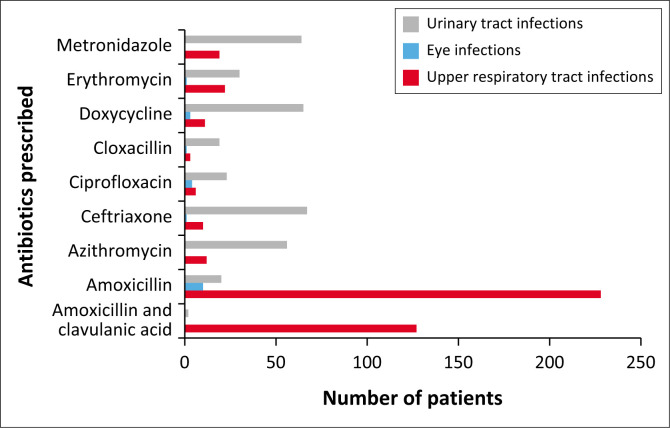
The overall top three diseases diagnosed among patients and the antibiotics most commonly prescribed for each.

In all, 898 medicine items were prescribed. Patients diagnosed with URTIs (*N* = 624) were prescribed amoxicillin, and amoxicillin and clavulanic acid combination (36.5% and 20.4%, respectively), whereas patients diagnosed with UTIs were prescribed ceftriaxone (10.7%), doxycycline (10.4%), metronidazole (10.3%) and erythromycin (4.8%). Patients diagnosed with eye infections received mainly amoxicillin (1.6%) and ciprofloxacin (0.6%) (Figure 2).

## Discussion

The overall mean prescribing of antibiotics in this study (1.48 (0.13) DDD/100 outpatients/day) was low compared to studies conducted in LMICs, such as in Ethiopia (5.31 DDD/100 outpatients/day),^14^ Siera Leone (5.53 DDD per 100 outpatients per day)^17^ and Syria (2.01 DDD per 100 outpatients per day).^18^ The variations in the seasons in which the studies were performed may have affected the differences between our study and the above-mentioned studies.^14,17,18^ Like Lesotho, Ethiopia has a STG and Essential Medicines List (EML) that promotes rational use of medicines,^19^ whereas Sierra Leone and Syria do not. Reports of inappropriate prescribing have, nevertheless, emerged in these countries.^20,21^

In this study, the Access group antibiotics (68.7% at PHCC-3 to 94% at PHCC-2) were prescribed more than the Watch group antibiotics (6.0% at PHCC-2 to 30.6% at PHCC-3), while the Reserve group and Not-Recommended antibiotics were not prescribed (Table 3). As the central store, the National Drug Service Organisation only provides medication to PHCCs based on the EML; medicine prescribing is, therefore, influenced by availability. In addition, more Access group antibiotics prescribed in the PHCCs in Lesotho could indicate adherence to the standard treatment guidelines, as most of the antibiotics in the Watch group are restricted from being used in PHCCs. This study also demonstrates adherence to the WHO recommendations advocating Access group antibiotics prescribing of above 60% of the total antibiotics.^10^ The findings of this study are further in agreement with a systematic review conducted in LMICs, which reported that countries such as Ethiopia, South Africa, Botswana, Nigeria, Zambia, Uganda and Kenya had more than 60% Access group antibiotics prescribed.^3^ Furthermore, a world prevalence survey indicated that the highest Access group antibiotic prescribing was reported in Guinea (66.7%), South Africa (61.9%) and Togo (59.8%).^22^ In addition, a point prevalence survey across six Tanzanian hospitals,^23^ a study conducted at the Ghana Police Hospital^24^ and a point prevalence survey across hospitals in Uganda^25^ all reported prescribing of Access group antibiotics above 60%. Our study showed that the amoxicillin and clavulanic acid combination, an Access group antibiotic, was only prescribed at two PHCCs whose prescribers were medical practitioners (Table 2). This may suggest the prescribers’ preferences. In this study, metronidazole was prescribed for UTI. According to the STGL, metronidazole is indicated for use in gynaecological conditions such as vaginal discharge^26^ and not for UTI; therefore, the antibiotic in this study was not prescribed according to protocol. Only one of the three PHCCs in this study had prescriptions for the Access group antibiotics below 80%. The staff that prescribes in this PHCC are registered nurse midwives. Therefore, a needs assessment is recommended among nurse midwives to determine their knowledge of prescribing antibiotics based on the AWaRe classification system and subsequent training based on the gap identified.

All the PHCCs in this study had a low prescribing frequency of antibiotics from the Watch group (Table 3), less than the WHO recommendation of less than 40%.^11^ The findings of this study concur with the results of a worldwide point prevalence survey in 69 countries where African countries such as Guinea, South Africa and Togo reported the lowest Watch prescribing percentages at 32.1%, 37.7%, and 39%, respectively.^22^ Despite the study’s low prescribing of Watch group antibiotics, the use of these medications needs to be closely monitored. Erythromycin (0.61 DDD/100 outpatients/day) and ceftriaxone (0.01 DDD/100 outpatients/day) were the most frequently prescribed antibiotics under the Watch group antibiotics (Table 2). Erythromycin and ceftriaxone were prescribed for UTI and vaginal discharge co-infections, as well as vaginal itchiness. According to the STGL, both ceftriaxone and erythromycin are indicated for use at hospitals and PHCCs to treat several infections, such as oral, eye and gynaecology conditions, and sexually transmitted infections (STIs).^26^ On the contrary, the WHO has classified ceftriaxone under the Watch group antibiotics and recommended it for use as second-line empiric treatment.^11^ When ceftriaxone is misused, multidrug-resistant bacteria can emerge, treatment costs will increase and adverse drug effects will occur.^27^ The usage of the Watch group antibiotics in this study demonstrates the need to strengthen or establish antibiotic stewardship (ABS) programmes in PHCCs in Maseru.

The results of this study further indicated that the Reserve and Not-Recommended group antibiotics were not prescribed in the three PHCCs. The results may have been influenced by the unavailability of Reserve group antibiotics because the EML restricts the PHCCs in terms of what to buy from the central medical stores. This is an indication of adherence to the STGL, as these antibiotics are only listed for use at the hospital level. It also indicates good practice, as Reserve group antibiotics should be used as the last resort treatment.^11^

In this study, the PHCCs had AW-I of 4.64, 16.49, and 2.24 for PHCC-1, PHCC-2, and PHCC–3, respectively (Table 3). In comparison, these indices are higher than in studies conducted in Ethiopia in 2019 (1.53),^14^ in Ghana in 2021 (1.2),^27^ and a point prevalence survey in 69 countries which reported AW-I in Guinea (2.1), South Africa (1.6) and Togo (1.5).^22^ Because the Watch group antibiotics are indicated for use as a second-line treatment option, the high AW-I in this study may indicate adherence to STGL. The prescribers may influence the difference in the AW-I between the PHCCs, as the AW-I was higher in PHCCs where medical practitioners are prescribers. There was a higher prescribing of amoxicillin in PHCC-2 than in PHCC-1 and PHCC-3, which is thought to have contributed to the high AW-I.

Antibiotic prescribing in this study varied among the PHCCs with the most prescribed antibiotics being amoxicillin and clavulanic acid combination (0.7 DDD/100 outpatients/day, 44.5% PHCC-1), amoxicillin (0.61 and 0.36 DDD/100 outpatients/day, 45.9% and 24.5% PHCC-2 and PHCC-3, respectively) and doxycycline (0.44 DDD/100 outpatients/day, 29.9% PHCC-3) as summarised in Table 2. Amoxicillin is the most widely used antibiotic,^28^ examples of which are studies conducted in community healthcare centres across South Africa.^3,29^ A high prescription of the combination of amoxicillin and clavulanic acid has also been reported in other countries, such as India,^30^ Syria^31^ and South Africa.^3,29^ In Lesotho, the combination of amoxicillin and clavulanic acid is indicated for use in several conditions, including respiratory infections, sores, infected wounds and peritonsillar abscesses, while doxycycline is indicated for STIs.^25^ The top three causes of death in Lesotho are human immunodeficiency virus/acquired immunodeficiency syndrome (HIV/AIDs), stroke, and respiratory infections.^32^ Human immunodeficiency virus infection has an overall prevalence of 22.7% in Lesotho, while a study performed in PHCCs in Lesotho reported an overall cumulative and comparative prevalence of 12.2% for STIs against 29.0% of people with HIV infection.^33,34^ The WHO recommends the interpretation of antibiotics used against the disease patterns^35^; it is assumed that the burden of URTIs and UTIs in the country influences the prescribing of the mentioned antibiotics in this study.

Lastly, this study reported that URTIs are the most diagnosed infection where antibiotics are used (Figure 1). This is in line with several other studies that indicate a high burden of URTI in primary healthcare, such as Namibia,^36^ Ghana,^37^ Ethiopia,^38^ rural Kenya,^39^ Tanzania^40^ and South Africa.^41^ The STGL states that URTIs, such as a common cold and influenza, are self-limiting; therefore, antibiotics should not be used.^26^ The use of antibiotics in this study for the management of URTIs is, therefore, against the STGL, indicating a need for the monitoring and optimisation of antibiotic use at the primary healthcare level. It should be noticed, however, that the study coincided with the coronavirus disease 2019 (COVID-19) pandemic, which could have influenced prescribing patterns. Nevertheless, healthcare professionals need to receive training on antibiotic use and prescribing.

### Limitations and strengths

The limitation of this study is that it only included three PHCCs in Maseru; the results may, therefore, not be generalised to all the PHCCs and hospitals in Lesotho. In addition, the data were collected for 1 month only, as a result, the seasonal variations in antibiotic prescribing could not be determined. The study also did not determine the appropriateness of antibiotic prescribing as laboratory tests were not performed. The study’s strength was that, to the best of our knowledge, it was the first of its sort to assess antibiotic prescribing using the WHO AWaRe classification in primary healthcare in Maseru, Lesotho; therefore, it serves as a baseline for monitoring antibiotic prescribing and establishing ABS programmes in PHCCs.

## Conclusion

In conclusion, the results indicate that the prescribing of Access group antibiotics is more frequent than the Watch group antibiotics. Reserve and Not-Recommended group antibiotics were not prescribed in this study. Even though Watch group antibiotics were low, antibiotics such as erythromycin, ceftriaxone and ciprofloxacin should be monitored as they are more prone to causing antimicrobial drug resistance. The establishment of ABS programmes should be encouraged and strengthened in primary healthcare, such as point-of-care tests, education of both patients and prescribers, and tracking and reporting of antibiotic prescribing. It is also recommended that this type of study be undertaken over a longer period to gain information on the influences of seasonal variation.

## References

[1] Klein EY, Van Boeckel TP, Martinez EM, et al. Global increase and geographic convergence in antibiotic consumption between 2000 and 2015. Proc Natl Acad Sci USA. 2018;115(15):E3463–E3470. 10.1073/pnas.1717295115 29581252 PMC5899442

[2] Centre for Disease Dynamics, Economics & Policy. State of the world’s antibiotics 2021: A global analysis of antimicrobial resistance and its drivers [homepage on the Internet]. 2021 [cited 2023 Jul 17]. Available from: https://onehealthtrust.org/publications/reports/the-state-of-the-worlds-antibiotic-in-2021/

[3] Sulis G, Adam P, Nafade V, et al. Antibiotic prescription practices in primary care in low- and middle-income countries: A systematic review and meta-analysis. PLoS Med. 2020;17(6):e1003139. 10.1371/journal.pmed.1003139 32544153 PMC7297306

[4] Mahmood RK, Gillani SW, Alzaabi MJ, et al. Evaluation of inappropriate antibiotic prescribing and management through pharmacist-led antimicrobial stewardship programmes: A meta-analysis of evidence. Eur J Hosp Pharm. 2021;29:2–7. 10.1136/ejhpharm-2021-002914 34848531 PMC8717790

[5] Wall S. Prevention of antibiotic resistance – An epidemiological scoping review to identify research categories and knowledge gaps. Global Health Action. 2019;12(1):1756191. 10.1080/16549716.2020.1756191 32475304 PMC7782542

[6] Ramachandran P, Rachuri NK, Martha S, et al. Implications of Overprescription of Antibiotics: A Cross-Sectional Study. J Pharm Bioallied Sci. 2019;11(2):S434–S437. 10.4103/JPBS.JPBS_62_19 31198382 PMC6555336

[7] Alabi ME, Essack SY. Antibiotic prescribing amongst South African general practitioners in private practice: An analysis of a health insurance database, JAC Antimicrob Resist. 2020;4(5):dlac101. 10.1093/jacamr/dlac101 PMC9524566 36196441

[8] Mthombeni TC, Burger JR, Lubbe MS, et al. Antibiotic prescribing to inpatients in Limpopo, South Africa: A multicentre point-prevalence survey. Antimicrob Resist Infect Control. 2023;12(1):103. 10.1186/s13756-023-01306-z 37717012 PMC10505321

[9] Ahiabu MA, Tersbøl BP, Biritwum R, Bygbjerg IC, Magnussen P. A retrospective audit of antibiotic prescriptions in primary health-care facilities in Eastern Region, Ghana. Health Policy Plan. 2016;31(2):250–258. https://doi.org/10.1093%2Fheapol%2Fczv048 26045328 10.1093/heapol/czv048 PMC4748131

[10] Adorka M, Mitonga HK, Lubbe M, et al. Assessment of the appropriateness of antibiotic prescriptions in Lesotho public hospitals: A novel methodology based on principles of antibiotic prescribing. J Public Health Afr. 2014;5(1):354. https://doi.org/10.4081%2Fjphia.2014.354 28299122 10.4081/jphia.2014.354 PMC5345467

[11] World Health Organization. AWaRe classification of antibiotics for evaluation and monitoring of use [homepage on the internet]. 2023 [cited 2023 Feb 17]. Available from: https://www.who.int/publications/i/item/WHO-MHP-HPS-EML-2023.04

[12] World Health Organization. Prioritization of pathogens to guide discovery, research and development of new antibiotics for drug-resistant bacterial infections, including tuberculosis [homepage on the Internet]. 2017 [cited 2023 Jan 15]. Available from: https://www.who.int/publications/i/item/WHO-EMP-IAU-2017.12

[13] World Population Review. Lesotho population [homepage on the Internet]. 2024 [cited 2023 Jun 18]. Available from: https://worldpopulationreview.com/countries/lesotho-population

[14] Melaku T, Gashaw M, Chelkeba L, et al. Evaluation of adult outpatient antibiotics use at Jimma Medical Centre (with defined daily doses for usage metrics). Infect Drug Resist. 2021;14:1649–1658. 10.2147/IDR.S293080 33953576 PMC8092616

[15] Hsia Y, Sharland M, Jackson C, et al. Consumption of oral antibiotic formulations for young children according to the WHO Access, Watch, Reserve (AWaRe) antibiotic groups: An analysis of sales data from 70 middle-income and high-income countries. Lancet Infect Dis. 2019;19(1):67–75. 10.1016/S1473-3099(18)30547-4 30522834

[16] De Bie S, Kaguelidou F, Verhamme KM, et al. Using prescription patterns in primary care to derive new quality indicators for childhood community antibiotic prescribing. Pediatr Infect Dis J. 2016;35(12):1317–1323. 10.1097/INF.0000000000001324 27626915

[17] Lakoh S, John-Cole V, Luke RDC, et al. Antibiotic use and consumption in Freetown, Sierra Leone: A baseline report of prescription stewardship in outpatient clinics of three tertiary hospitals. IJID Reg. 2023;7:43–51. 10.1016/j.ijregi.2023.02.004 37038468 PMC10082370

[18] Aljadeeah S, Wirtz VJ, Nagel E. Outpatient antibiotic dispensing for the population with government health insurance in Syria in 2018–2019. Antibiotics (Basel). 2020;9(9):570. 10.3390/antibiotics9090570 32887446 PMC7559287

[19] Ministry of Health Ethiopia. Standard treatment guidelines [homepage on the Internet]. 2021 [cited 2023 Feb 20]. Available from: https://www.slideshare.net/slideshow/stg-2021pdf/252255886

[20] Al Sous MM, Al Houri HN, Safiah MH, Alazrak SO, et al. Antibiotic prescription patterns for acute upper respiratory tract infections in an outpatient population with health insurance in Syria – A retrospective cross-sectional study. IJID Reg. 2023;1(7):66–71. 10.1016/j.ijregi.2023.02.010 PMC10064427 37009572

[21] Kabba JA, Tadesse N, James PB, et al. Knowledge, attitude and antibiotic prescribing patterns of medical doctors providing free healthcare in the outpatient departments of public hospitals in Sierra Leone: A national cross-sectional study. TRSTMH. 2020;114(6):448–458. 10.1093/trstmh/trz137 31999320

[22] Pauwels I, Versporten A, Drapier A. The Global-PPS network, Hospital antibiotic prescribing patterns in adult patients according to the WHO Access, Watch and Reserve classification (AWaRe): Results from a worldwide point prevalence survey in 69 countries, J Antimicrob Chemother. 2021;76(6):1614–1624. 10.1093/jac/dkab050 33822971 PMC8120336

[23] Seni J, Mapunjo SG, Wittenauer R, et al. Antimicrobial use across six referral hospitals in Tanzania: A point prevalence survey. BMJ Open. 2020;10:e042819. 10.1136/bmjopen-2020-042819 PMC7745526 33323448

[24] Darkwah TO, Afriyie DK, Sneddon J, et al. Assessment of prescribing patterns of antibiotics using National Treatment Guidelines and World Health Organization prescribing indicators at the Ghana Police Hospital: A pilot study. Pan Afr Med J. 2021;39:222. 10.11604/pamj.2021.39.222.29569 34630834 PMC8486929

[25] Kiggundu R, Wittenauer R, Waswa JP. Point prevalence survey of antibiotic use across 13 hospitals in Uganda. Antibiotics (Basel). 2022;11(2):199. 10.3390/antibiotics11020199 35203802 PMC8868487

[26] Ministry of Health. Standard treatment guidelines for Lesotho [homepage on the Internet]. 2022 [cited 2023 Jul 20]. Available from: https://static1.squarespace.com/static/60bdea12fbad083dee4ffa2c/t/651fb589cf17c5449fc9ac66/1696576912929/STG+LESOTHO+Feb+2023+%281%29.pdf

[27] Kutyabami P, Munanura EI, Kalidi R, et al. Evaluation of the clinical use of Ceftriaxone among in-patients in selected health facilities in Uganda. Antibiotics (Basel). 2021;10(7):779. 10.3390/antibiotics11020199 34202391 PMC8300672

[28] StatPearls. Amoxicillin [homepage on the Internet]. 2023 [cited 2023 Jan 15]. Available from: https://www.ncbi.nlm.nih.gov/books/NBK482250/

[29] Lagarde M, Blaauw D. Levels and determinants of overprescribing of antibiotics in the public and private primary care sectors in South Africa. BMJ Global Health. 2023;8:e012374. 10.1136/bmjgh-2023-012374 PMC10391785 37524502

[30] Adedapo AD, Akunne OO. Patterns of antimicrobials prescribed to patients admitted to a tertiary care hospital: A prescription quality audit. Cureus. 2021;13(6):e15896. https://doi.org/10.7759%2Fcureus.15896 34322343 10.7759/cureus.15896 PMC8309689

[31] Tomas A, Aljadeeah S. The overlooked issue of outpatient combination antibiotic prescribing in low-and middle-income countries: An example from Syria. Antibiotics (Basel). 2022;11(1):74. 10.3390/antibiotics11010074 35052951 PMC8772973

[32] World Health Organization data. Lesotho [homepage on the Internet]. 2022 [cited 2023 May 01]. Available from: https://data.who.int/countries/426

[33] Endline assessment, reporting and documentation of the integrated HIV/AIDS-nutrition project in Lesotho United Nations Lesotho [homepage on the Internet]. 2023 [cited 2023 Nov 13]. Available from: https://lesotho.un.org/en/217138-endline-assessment-reporting-and-documentation-integrated-hivaids-nutrition-project-lesotho

[34] Xavier LN, Mokgatle MM, Oguntibeju OO. Prevalence of sexually transmitted infections and related sexual behaviour among pregnant women 18–49 years old attending antenatal clinic at a primary health care in Maseru, Lesotho. TOPHJ. 2023;16:e18749445255982. 10.2174/0118749445255982231031051957

[35] WHO report on surveillance of antibiotic consumption: 2016–2018 early implementation [homepage on the Internet]. 2018 [cited 2023 Feb 10]. Global Antimicrobial Resistance Surveillance System (GLASS). Available from: https://www.who.int/publications/i/item/9789241514880

[36] Niaz Q, Godman B, Massele A, et al. Validity of World Health Organisation prescribing indicators in Namibia’s primary healthcare: Findings and implications. Int J Qual Health Care. 2019;31(5):338–345. 10.1093/intqhc/mzy172 30169688

[37] Prah J, Kizzie-Hayford J, Walker E, et al. Antibiotic prescription pattern in a Ghanaian primary health care facility. Pan Afr Med J. 2017;28:214. 10.11604/pamj.2017.28.214.13940 29610652 PMC5878858

[38] Yimenu DK, Emam A, Elemineh E, et al. Assessment of antibiotic prescribing patterns at outpatient pharmacy using World Health Organization prescribing indicators. J Prim Care Community Health. 2021;10:2150132719886942. 10.1177/2150132719886942 PMC6836303 31690162

[39] Nyamu N, Mbatia F, Van den Hombergh P, et al. Burden of upper respiratory tract infections in primary care facilities and excessive antimicrobial over-prescription: A community-oriented primary care project in rural Kenya. Afr J Prim Health Care Fam Med. 2021;13(1):e1–e4. https://doi.org/10.4102%2Fphcfm.v13i1.3107 10.4102/phcfm.v13i1.3107 PMC8661269 34879697

[40] Kilipamwambu A, Bwire GM, Myemba DT, et al. WHO/INRUD core prescribing indicators and antibiotic utilization patterns among primary health care facilities in Ilala district, Tanzania. JAC Antimicrob Resist. 2021;3(2):dlab049. 10.1093/jacamr/dlab049 34223117 PMC8210294

[41] Chigome A, Ramdas N, Skosana P, et al. A narrative review of antibiotic prescribing practices in primary care settings in South Africa and potential ways forward to reduce antimicrobial resistance. Antibiotics (Basel). 2023;12(10):1540. 10.3390/antibiotics12101540 37887241 PMC10604704

